# Possible Alterations in β-Synuclein, the Non-Amyloidogenic Homologue of α-Synuclein, during Progression of Sporadic α-Synucleinopathies

**DOI:** 10.3390/ijms130911584

**Published:** 2012-09-14

**Authors:** Masayo Fujita, Akio Sekigawa, Kazunari Sekiyama, Yoshiki Takamatsu, Makoto Hashimoto

**Affiliations:** Division of Sensory and Motor Systems, Tokyo Metropolitan Institute of Medical Science, 2-1-6 Kamikitazawa Setagaya-ku Tokyo 156-0057, Japan; E-Mails: sekigawa-ak@igakuken.or.jp (A.S.); sekiyama-kz@igakuken.or.jp (K.S.); takamatsu-ys@igakuken.or.jp (Y.T.)

**Keywords:** α-synucleinopathies, α-synuclein, β-synuclein, dementia with Lewy bodies, toxic gain of function

## Abstract

α-Synucleinopathies are neurodegenerative disorders that are characterized by progressive decline of motor and non-motor dysfunctions. α-Synuclein (αS) has been shown to play a causative role in neurodegeneration, but the pathogenic mechanisms are still unclear. Thus, there are no radical therapies that can halt or reverse the disease’s progression. β-Synuclein (βS), the non-amyloidogenic homologue of αS, ameliorates the neurodegeneration phenotype of αS in transgenic (tg) mouse models, as well as in cell free and cell culture systems, which suggests that βS might be a negative regulator of neurodegeneration caused by αS, and that “loss of function” of βS might be involved in progression of α-synucleinopathies. Alternatively, it is possible that “toxic gain of function” of wild type βS occurs during the pathogenesis of sporadic α-synucleinopathies, since tg mice expressing dementia with Lewy bodies-linked P123H βS develop progressive neurodegeneration phenotypes, such as axonal pathology and dementia. In this short review, we emphasize the aspects of “toxic gain of function” of wild type βS during the pathogenesis of sporadic α-synucleinopathies.

## 1. Introduction

β-Synuclein (βS) is a presynaptic protein of unknown function that was originally isolated as a phosphoprotein from bovine brain by Nakajo and colleagues in 1993 [1–3]. During the same period, two structurally related proteins; α-synuclein (αS) and γ-synuclein (γS), were also found independently by several groups. After extensive characterization of the synuclein family of peptides, it is now well established that αS plays a causative role in stimulation of α-synucleinopathies, including Parkinson’s disease (PD), dementia with Lewy bodies (DLB), multiple system atrophy, neurodegeneration with brain iron accumulation, type 1 (formerly known as Hallervorden-Spatz disease), and the Lewy body variant of Alzheimer’s disease [4,5], while γS is involved in tumor progression and metastasis of cancers such as breast cancer, ovarian tumor and brain tumors [6].

In contrast to the other two members, the pathogenic role of βS has been elusive. In this regard, we previously showed that the neurodegeneration phenotype of the αS transgenic (tg) was ameliorated by cross-breeding with a tg mouse expressing wild type βS, and proposed that non-amyloidogenic βS might negatively regulate neurodegeneration caused by amyloidogenic αS [7]. More recently, we showed that tg mice expressing DLB-linked P123H βS develop progressive neurodegeneration, as characterized by axonal pathology and memory disorder, suggesting that the DLB-linked mutant βS is pathogenic [8]. This raises the question of how these apparently opposite effects of wild type βS and DLB-linked mutant βS in tg mice brain should be interpreted. Is it sufficient to conclude that wild type βS is simply neuroprotective and that this is distinct from the stimulatory effect of neurodegeneration by DLB-linked mutant βS?

In this review, we propose a new perspective that “toxic gain of function” of wild type βS might be a critical event during the pathogenesis of sporadic α-synucleinopathies. This scenario may permit development of a comprehensive model of βS actions in the brain in α-synucleinopathies, including findings from our studies using tg mice and reports by other laboratories.

## 2. βS with a DLB-Linked Mutation Shows “Gain of Function” in α-Synucleinopathies

A series of studies in our laboratory have shown that βS with a DLB-linked mutation is an aggregate-prone protein and that “toxic gain of function” of mutant βS occurs in cell-free, cellular and tg experimental models.

Two missense mutations of βS were found in unrelated DLB patient pedigrees in 2004. A valine to methionine substitution at position 70 (V70M) was found in a sporadic DLB case in Japan, while a proline to histidine mutation at position 123 (P123H) was identified in a familial DLB pedigree in Seattle. Since P123H βS was inherited as an autosomal dominant trait, the βS gene mutations might have caused a toxic gain of function, similarly to cases with αS mutations. However, there was no pathological evidence of βS in autopsy brains of patients with the P123H βS mutation, including the absence of immunoreactivity of βS in Lewy bodies, and no aggregated form of βS in biochemical analysis. Thus, it was proposed that the mutated forms of βS might have lost the protective function of wild type βS.

To understand the role of the DLB-linked mutant form of βS in neurodegeneration more clearly, we analyzed P123H and V70M recombinant βS proteins in a cell-free system, cell culture, and transgenic (tg) mice. Under cell-free conditions, both proteins were prone to aggregation and acted synergistically with αS to stimulate protein aggregation [9]. In B103 neuroblastoma cells expressing P123H or V70M βS, abnormal lysosomal inclusions were formed, in which mutant βS accumulated due to impairment of the autophagy-lysosome pathway [9]. Furthermore, the number of lysosomal inclusions was markedly increased by coexpression of αS and mutant βS [9]. Taken together, these results suggest that both mutant forms of βS could themselves be pathogenic and might act synergistically with αS.

This view was further supported by experiments in a tg mouse expressing P123H βS under the Thy-1 promoter. These mice displayed several neuropathological abnormalities, including formation of P123H βS-immunoreactive axonal swellings in basal ganglia and extensive astrogliosis in various brain regions, including the hippocampus, cerebral cortex and basal ganglia, indicating that P123H βS is pathogenic *in vivo* [8]. Strikingly, P123H βS tg mice also showed significant memory dysfunction at a relative early age (~6 months), while motor dysfunction was apparent in a later stage (over 12 months) [8]. These behavioral features were distinct from tg mice expressing Thy-1 promoter-driven αS, which showed significant motor dysfunction at an earlier age (~4 months). The discovery of βS mutations in DLB patients suggests that βS may be involved more specifically in memory functions, compared to αS. Consistent with this idea, βS was recently shown to be a strain-independent hippocampal protein that is upregulated during the Morris water maze test [8]. Thus, these results suggest that the pathological effects of βS might be distinct from those of αS.

Further cross-breeding experiments revealed that bigenic mice expressing αS and P123H βS exhibited greatly enhanced neurodegeneration phenotypes, including earlier appearance of axonal swelling, a decreased level of dopamine in the striatum, motor dysfunction, neuroinflammation, and neuronal cell death [8]. These results indicate that αS and P123H βS may synergistically enhance the neuropathological effect of each protein.

Taken together, the analyses of our P123H βS tg mice suggest that P123H βS is pathogenic through a “gain of function” mechanism and may cooperate with pathogenic αS to stimulate neurodegeneration in mouse brain, indicating a causative role of P123H βS in the pathogenesis of familial DLB.

## 3. Does “Loss of Function” Explain the Role of Wild Type βS in Sporadic α-Synucleinopathies?

We have shown that wild type βS is protective, whereas βS with a DLB-linked mutation is stimulatory for neurodegeneration. These results prompted us to consider if wild type βS might be involved in the pathogenesis of sporadic DLB and other α-synucleinopathies. To date, several studies have shown that “loss of function” of wild type βS could contribute to the pathogenesis of α-synucleinopathies.

Masliah and colleagues used a RNA-protection assay that was previously used to show that the ratio of βS to αS at the mRNA level was significantly decreased in diseased brains, including PD, DLB and AD brains, compared to healthy brains [10]. These results are intriguing because decreased expression of βS may lead to relative loss of protective functions of βS against neurotoxicity caused by αS. Furthermore, it is possible that downregulation of βS could occur not only in α-synucleinopathies, but also in other types of neurodegenerative disease. More recently, Ariza and colleagues performed a real-time polymerase chain reaction analysis to show that βS mRNA expression was significantly decreased in cortical areas of pure DLB pathology and in the clinical phenotype of DLB, but not in cases with diffuse Lewy body pathology and concomitant AD pathology or in the clinical phenotype of PD (PD with dementia) [11]. These results suggest the existence of a specific molecular subtype of DLB characterized by a strong decrease of βS.

Taken together, downregulation of βS mRNA in autopsy brains of α-synucleinopathies suggest that “loss of function” of βS occurs in pathogenesis of sporadic α-synucleinopathies. In this regard, future studies are warranted to examine this possibility at the protein level, since expression levels of proteins are not directly reflected by the mRNA level and may be affected by the clearance of protein. This is critical in the aging process and under disease conditions in which protein degradation systems, such as the proteasome and autophagy-lysosome pathways, are compromised.

## 4. Rebutting Genetic Reports Showing a Negative Association of Wild Type βS with Sporadic α-Synucleinopathies

Several studies have indicated that synuclein gene polymorphisms could be involved in the onset of α-synucleinopathies. Among the synuclein genes, αS and γS gene polymorphisms and SNPs have been related to disease. In contrast, few relationships have been proposed between βS gene polymorphisms and the onset of α-synucleinopathies, although one SNCB (βS) SNP (rs1352303) has been associated with delayed age at onset of PD in women, despite SNCB not being a susceptibility gene for PD [12]. Another study indicated that αS and γS genes have particular effects on the risk of developing diffuse Lewy body disease (DLBD), whereas variants of the βS gene showed the least evidence of an association with DLBD [13].

Based on these findings, it may be argued that βS is not related to the pathogenesis of α-synucleinopathies, or at least that βS does not obtain a “toxic gain of function”, distinct from αS and γS. However, it is important to remember that diagnosis of DLBD is based on the presence of Lewy bodies. It has been well characterized that formation of Lewy bodies is a protective reaction in surviving neurons, especially during an advanced stage of neurodegeneration. Since βS is involved in axonal pathology but not in Lewy body pathology, it is reasonable that SNPs of the βS gene are not associated with DLBD. In this context, it is intriguing to speculate that SNCB SNPs may correlate with the extent of axonopathy in DLBD.

## 5. “Toxic Gain of Function” Scenario of Wild Type βS

**Scheme 1:**
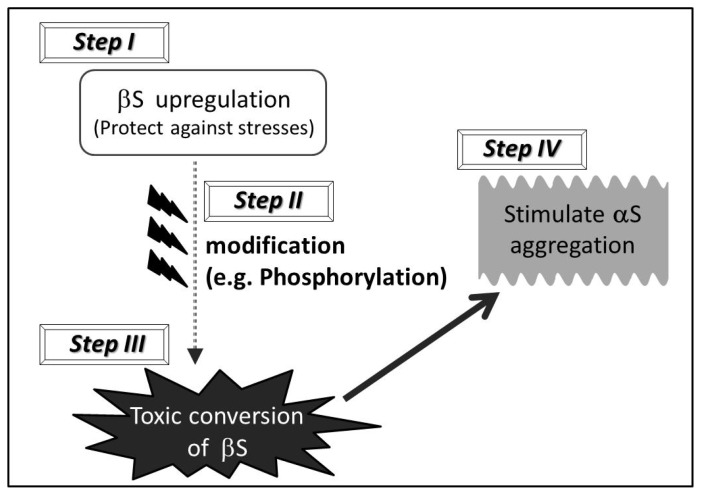
Schematic hypothesis of a multiple-step mechanism through which βS may stimulate the pathogenesis of sporadic α-synucleinopathies.

At present, it is unknown whether “toxic gain of function” of wild type βS is indeed the case for the pathogenesis of sporadic α-synucleinopathies. In this regard, we propose a hypothetical multiple-step mechanism (steps I–IV) through which wild type βS might stimulate the axonal pathology of α-synucleinopathies in human brains (Scheme 1). In the initial phase, βS might be accumulated in the presynapse (or distal axon) due to genetic factors, environmental factors, and aging. In particular, given the neuroprotective effect of wild type βS, βS might be upregulated to protect against increasing stresses, such as oxidative stress and chronic inflammation, during the course of aging (step I). Accumulated βS might then gradually undergo posttranslational modifications, such as phosphorylation and glycosylation, particularly in the *C*-terminal region (step II). As a consequence, a small amount of wild type βS with extensive modifications might adopt altered structures (e.g., toxic oligomers, protofibrils) due to aberrant regulation of the *C*-terminal region. This step may be regarded as “transformation” of βS from a neuroprotective to a neurodegenerative molecule (step III). Then, wild type βS with an altered structure may sequester αS, further stimulating the process of αS aggregation. Once aggregation of αS starts, amyloidogenesis of αS might proceed even in the absence of βS (step IV). Overall, alteration of βS might be involved in the early stage of the amyloidogenesis of αS, whereby altered βS may interact with αS, resulting in initial seeding of aggregated αS and leading to promotion of αS pathology.

## 6. Evidence that Wild Type βS Exhibits “Gain of Function” Properties in α-Synucleinopathies

According to the “gain of function” scenario, wild type βS might be accumulated in the initial stage of sporadic α-synucleinopathies (step I). Indeed, accumulation of wild type βS has been observed in various aspects of neurodegeneration. For instance, βS was found to be abnormally concentrated in dystrophic neurites in the hippocampal region in brains from PD and DLB patients [14]. Similarly, βS was detected in spheroids, but not in Lewy body-like or glial inclusions, in neurodegeneration with brain iron accumulation, type 1 [15]. These results suggest that accumulation of wild type βS in the axonal pathology could be an early step in sporadic α-synucleinopathies.

Accumulated wild type βS might be subjected to posttranslational modifications (step II). Indeed, several serine/threonine residues of βS are constitutively phosphorylated [1]. In particular, it is of interest to determine if phosphorylation of S118 confers enhanced aggregation properties on βS, as is the case for phosphorylation of S129 of αS [16]. In a similar context, tyrosine residues Y119, Y127 and Y130 of βS, which are potential targets of non-receptor tyrosine kinases (e.g., src family kinases), might affect aggregation of βS, since aggregation of αS is negatively regulated by phosphorylation of the corresponding Y125, Y133 and Y136 [17,18]. Furthermore, it is of note that βS is a major *O*-glycosylated protein in the presynapse of rat brain [19]. Assuming that the serine residues compete for glycosylation and phosphorylation, glycosylation may also be involved in aberrant posttranslational modifications of βS during aging or due to environmental factors. Finally, it is also of note that a polyproline II (PPII) helix is clustered in the *C*-terminal region (EPLXEPLXEPE motif: 105–115) of βS and that posttranslational modifications and P123H mutation could affect this important structure [20]. Given the role of PPII helices in protein-protein interactions through binding with SH3 domains [21], it is possible that the PPII domain might be a locally important regulatory domain for the aggregation properties of βS.

To date, fibril formation of wild type βS has not been reported *in vivo* or in cell cultures. However, in a cell-free system wild type βS was induced to form amyloid fibrils in the presence of specific metal ions and glycosaminoglycan macromolecular crowding agents [22]. Thus, aggregation and fibril formation of wild type βS may be possible *in vivo* (step III). Such aggregation may also involve effects of a minor transcript of βS. In the SNCA gene, SNCA112 and SNCA98 are aggregation-prone isoforms and SNCA126 is a potentially protective isoform [23,24]. Similarly to SNCA, Ariza and colleagues have shown the presence of novel minor splicing variants of SNCB, including variants lacking exon 4 (a homolog of SNCA126) and exon 6 (a homolog of SNCA112) [25]. These minor isoforms have not been detected at the protein level due to their extremely low expression compared to major isoforms, but it is an intriguing possibility that dysregulation of the products of minor transcripts, especially the variant lacking exon 6, might permit formation of aggregates that act as seeds that trigger αS aggregation.

## 7. Towards Novel Therapeutic Strategies

Since several studies have indicated decreased βS expression in some brain regions in α-synucleinopathies, compensation for decreased expression of βS may be an effective strategy. For instance, it was previously shown that viral delivery of βS to the brains of αS tg mice decreased αS aggregation in the brains of the treated mice [26]. In this study, expression of full length of βS was induced in the brain of αS tg mice using a lentivirus vector. In addition, short peptides derived from βS were effective for prevention of αS aggregation in the brain of αS tg mice. More recently, it was shown that locomotor activity and accumulation of A53T αS in the brain of a drosophila model of PD expressing A53T αS was significantly ameliorated when the flies were fed with retro-inverso βS peptides [27]. Thus, if “loss of function” occurs, then replenishment of βS is a potential therapeutic strategy.

On the other hand, βS might become pathological by gene mutation (e.g., P123H) or through other unknown modifications, as described in this review. In addition, considering the potential role of some βS isoforms in several brain regions in α-synucleinopathies, the involvement of βS in these diseases might be explained by “gain of function”. If some βS molecules are pathogenic, then it is reasonable to target the pathogenic βS. Vaccination with αS is effective for decreasing αS at the protein level [28], and recent studies indicate that pathological αS may transmit to other cells and result in progression of α-synucleinopathies [29]. Thus, vaccination of αS may contribute to reduction of extracellular αS and prevent progression of α-synucleinopathies. In a similar context, pathogenic βS could also be removed by vaccination. Whether cytoplasmic βS is released into the extracellular space is currently unknown; however, it may be possible to decrease the intracellular βS level using vaccination, similarly to αS. Thus, if “gain of function” occurs, mixed vaccination with αS and βS could be a possible therapeutic strategy.

Finally, both “loss of function” and “gain of function” of wild type βS might occur in the same brain. In such a situation, the therapeutic strategy will become more complicated. Although vaccination with βS may be effective for removal of pathogenic βS, it may be difficult to control the expression level of βS throughout the brain. To establish novel therapies that are satisfactory for both compensation and vaccination, technological innovations are required to achieve region-specific regulation of βS expression in α-synucleinopathies.

## 8. Conclusion

In conclusion, we assume that not only “loss of function” of βS but also “toxic gain of function” of βS may be involved in progression of sporadic α-synucleinopathies. In this context, a better understanding of the role of wild type βS in the pathogenesis of sporadic α-synucleinopathies is critical for development of new avenues of therapy for these devastating diseases.
